# Radio-Iodide Treatment: From Molecular Aspects to the Clinical View

**DOI:** 10.3390/cancers13050995

**Published:** 2021-02-27

**Authors:** Antonio De la Vieja, Garcilaso Riesco-Eizaguirre

**Affiliations:** 1 Endocrine Tumors Unit (Unidad Funcional de Investigación en Enfermedades Endocrinas (UFIEC), Instituto de Salud Carlos III (ISCIII), Majadahonda, 28220 Madrid, Spain; 2 Centro de Investigación Biomédica en Red de Cáncer (CIBERONC), Instituto de Salud Carlos III (ISCIII), 28029 Madrid, Spain; griesco@iib.uam.es; 3 Departamento de Endocrinología y Nutrición, Hospital Universitario de Móstoles, 28935 Madrid, Spain; 4 Molecular Endocrinology Group, Faculty of Medicine, Universidad Francisco de Vitoria, 28223 Madrid, Spain

**Keywords:** radio-iodide treatment, thyroid cancer, sodium/iodide symporter (NIS), thyroid hormonal deprivation, recombinant human TSH, theragnostic, stunning, differentiated thyroid cancer, radio-iodine-refractory thyroid cancer, adjuvant therapy

## Abstract

**Simple Summary:**

This year marks the 80th commemoration of the first time that radio-iodide treatment (RAI) was used. RAI is one of the most effective targeted internal radiation anticancer therapies ever devised and it has been used for many decades, however, a thorough understanding of the underlying molecular mechanisms involved could greatly improve the success of this therapy. This is an in-depth innovative review focusing on the molecular mechanisms underlying radio-iodide therapy in thyroid cancer and how the alteration of these mechanisms affects the results in the clinic.

**Abstract:**

Thyroid radio-iodide therapy (RAI) is one of the oldest known and used targeted therapies. In thyroid cancer, it has been used for more than eight decades and is still being used to improve thyroid tumor treatment to eliminate remnants after thyroid surgery, and tumor metastases. Knowledge at the molecular level of the genes/proteins involved in the process has led to improvements in therapy, both from the point of view of when, how much, and how to use the therapy according to tumor type. The effectiveness of this therapy has spread into other types of targeted therapies, and this has made sodium/iodide symporter (NIS) one of the favorite theragnostic tools. Here we focus on describing the molecular mechanisms involved in radio-iodide therapy and how the alteration of these mechanisms in thyroid tumor progression affects the diagnosis and results of therapy in the clinic. We analyze basic questions when facing treatment, such as: (1) how the incorporation of radioiodine in normal, tumor, and metastatic thyroid cells occurs and how it is regulated; (2) the pros and cons of thyroid hormonal deprivation vs. recombinant human Thyroid Stimulating Hormone (rhTSH) in radioiodine residence time, treatment efficacy, thyroglobulin levels and organification, and its influence on diagnostic imaging tests and metastasis treatment; and (3) the effect of stunning and the possible causes. We discuss the possible incorporation of massive sequencing data into clinical practice, and we conclude with a socioeconomical and clinical vision of the above aspects.

## 1. Radioactive Iodide (RAI) Therapy

Radioactive iodide (RAI) therapy has been a treatment option for patients with benign and malignant thyroid disease since the 1940s [1,2]. This post-surgical treatment after thyroidectomy is the oldest targeted therapy and contributes significantly to differentiated thyroid cancer (DTC) patients’ life expectancy. The potential secondary effects such as damage to salivary glands or tear ducts, or soreness and swelling in glands, are generally temporary and do not outweigh the benefits for thyroid malignancies. RAI is also used in benign thyroid diseases such as hyperthyroidism [3].

**Figure 1:**
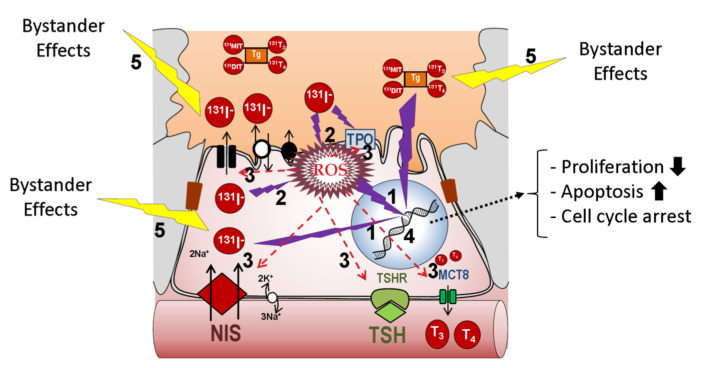
Putative effects of radio-iodide in the cell. Two types of effects can be experienced: direct and indirect. Four main types of damage can occur as a result of direct effects: (1) direct DNA damage such as single-strand and double-strand breaks, (2) increased ROS, (3) inactivation of DNA repair proteins to compensate for elevated ROS, (4) elevated ROS-mediated protein inactivation of proteins directly implicated in iodide transport, such as NIS, or in general thyroid differentiation (TPO, Tg, Duox2, TSH-R, Pendrin, etc.). Indirect non-targeted effects of radiation (5) occur in the surrounding cells as a result of bystander and abscopal effects. In any case, damage intensity would depend on radioiodine concentration and subcellular localization. Abbreviations: NIS: sodium/iodide symporter; TPO: thyroid peroxidase; Tg: thyroglobulin; TSH-R: Thyroid Stimulating Hormone receptor; ROS: reactive oxygen species; Duox2: Dual oxidase 2; MCT8: Monocarboxylate transporter 8.

RAI is based on radiation capacity. In the case of ^131^I, high energy nuclear electron emissions are used to destroy target cells (Table 1) (Figure 1). Ionizing radiation leads to DNA damage, which is primarily caused by both the direct and indirect effects of radiation. This leads to molecular damage such as single-strand breaks, double-strand breaks, base damage, and DNA–protein cross links [4]. In the direct effects, ^131^I significantly inhibits cell proliferation, enhances cell apoptosis by downregulating the effector of cell cycle checkpoint *Bcl2* gene, and promotes cell cycle arrest by upregulating the B-cell translocation gene 2-mediated activation of JNK/NF-κB pathways [5]. The sodium/iodide symporter (NIS) is also susceptible to DNA damage involving ataxia telangiectasia mutated kinase (ATM)-mediated mechanisms [6]. Indirect effects of radiation occur in the surrounding cells through non-targeted (bystander and abscopal) effects [7].

## 2. Iodide Accumulation in Normal, Tumor, and Metastatic Thyroid Cells

RAI therapy is a broad term that encompasses three different treatments associated with the administered activity of ^131^I [8]. The first, remnant ablation, is where ^131^I is given to destroy normal residual functioning thyroid tissue after surgery. This increases the sensitivity of detection of putative locoregional and/or metastatic disease on whole-body scans, maximizes therapeutic effects, and identifies additional sites of tumor cells. The second, adjuvant treatment, is where ^131^I is used to destroy unknown microscopic thyroid tumor cells and potentially decrease the chance of recurrence and patient mortality. Finally, RAI treatment of known disease is where ^131^I is given to destroy locoregional and distant metastasis to cure patients more efficiently, to reduce recurrence and mortality, and in cases of palliative care. RAI therapy is usually recommended for patients who have DTC. There are still issues to resolve regarding the therapeutic use of ^131^I for DTC once implementing RAI in clinical practice is considered [9]. Among others, these include the best method of preparation (thyroid hormone (TH) deprivation vs. recombinant human Thyroid Stimulating Hormone (rhTSH)), the amount of ^131^I used (low vs. high doses) depending on tumor risk stratification, assessment of post-operative disease status, precise definition of successful therapy, the radioisotopes chosen in diagnosis to avoid stunning, the use of personalized dosimetry, the management of refractory RAI cases, and the evaluation of putative side effects in the risk–benefit ratio of RAI to optimize the decision-making process. However, RAI therapy has provided undoubted benefits for DTC patients, and its success in their treatment has turned RAI into a potential therapeutic tool for other extra-thyroid tumors that express NIS [10,11,12,13]. Today, RAI through NIS is one of the favored theragnostic tools in gene therapies [13,14,15,16,17]. Nevertheless, RAI therapy success essentially depends on the capacity of the cell to accumulate radioactive iodide.

One of the main questions when approaching treatment is how radioiodine is incorporated into the tumor cell. To understand this process, it is necessary to describe the incorporation of iodine by NIS into a thyroid cell in normal physiology. The main role of iodine in metabolism is the synthesis of TH, which occurs in the thyroid gland [18,19]. Therefore, it is essential to know the molecular mechanisms involved in order to understand the incorporation of stable iodine (^127^I^−^), its radioactive isotopes (^123^I^−^, ^124^I^−^, ^125^I^−^, ^131^I^−^), and other thyroid-accumulated radioisotopes such as ^99m^TcO_4_^−^, ^188^ReO_4_^−^, ^18^F-FDG, and ^18^F-TFB which are currently used in clinical medicine (Table 1) [20].

### 2.1. Physiology of the Thyroid Epithelial Cell

**Figure 2:**
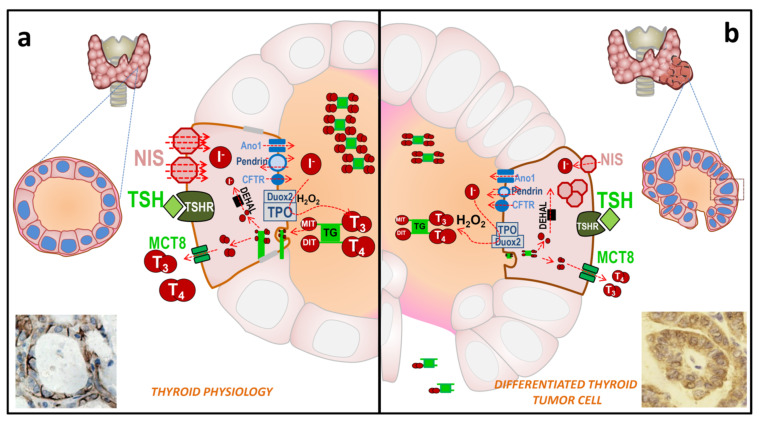
Schematic representation of the biosynthesis of thyroid hormones (TH) in normal and differentiated thyroid cancer (DTC) thyroid tissue. (**a**) Physiology of a thyroid cell. The thyroid follicle is made up of epithelial cells. The basolateral membrane is in contact with the blood stream, where iodide from the diet, or iodide recycled from dehalogenation in peripheral tissues, arrives. Iodide is transported against its concentration gradient by the plasma membrane protein sodium/iodide symporter (NIS) (red diamonds) thanks to the sodium gradient provided by the sodium and potassium-ATPase. NIS can concentrate more than 40–100 times the concentration of iodide in the blood. The iodide goes to the apical membrane side of the cell, essentially by concentration gradient, and is then transported to the colloid by means of different transporters (Anoctamin 1 (Ano1/TMEM16A), Pendrin and cystic fibrosis transmembrane conductance regulator (CFTR) located in the apical membrane of the thyroid follicle cell. The Duox2 enzyme generates H_2_O_2_, which is used by TPO to oxidize iodide in the TG molecule (organification), forming iodotyrosine residues (MIT and DIT). TPO itself couples two residues of MIT and/or DIT to synthetize the thyroid hormones (TH) T3 and T4, although they are still bound to TG. Iodinated TG (TG-I), as iodotyrosine or iodotyronine residues, accumulates in the colloid until there is further need for TH. When TH is required, TG is internalized into the cytoplasm, proteolyzed, and the iodinated residues are released. The iodine from the MIT and DIT residues is recycled by the Iodotyrosine dehalogenase 1 (DEHAL) enzyme. T3 and T4 are transported to the bloodstream by the MCT8 membrane protein. TSH, through its receptor on the basolateral membrane (TSH-R), is the master hormone that regulates most of the indicated processes at different levels, in addition to cell proliferation and growth. Immunohistochemistry shows the expression of NIS in the basolateral membrane of a non-anaplastic thyroid. (**b**) Thyroid tumor cell physiology in differentiated thyroid cancer (DTC). In tumor cells, the follicular structure is not always well preserved, and TG can be released into the bloodstream. The expression of key TH synthesis proteins is reduced (TSH-R, NIS, TPO, and pendrin) or delocalized from the place where they are functional, which prevents correct TH synthesis. Furthermore, Duox2 is usually not diminished, so the production of H_2_O_2_ does not decrease, which may lead to an increase in ROS that alter the synthesis and degradation of cell mechanisms. Immunohistochemistry shows the expression of NIS localized both at the basolateral membrane and in the cytoplasm in DTC tissue.

The key molecule involved in the entry of iodine into a thyroid follicle cell is the NIS protein [13,21,22,23,24] (Figure 2a). NIS is expressed on the basolateral membrane of the thyroid follicular cell to allow the transport of iodine from the bloodstream into the cell cytoplasm. NIS transports iodine against its concentration gradient using the favorable sodium gradient since the concentration of Na^+^ is much higher in the blood than inside cells. NIS transports not only iodine, but also other ions such as ClO_4_^−^ > ReO_4_^−^ > I^−^ ≥ SeCN^−^ ≥ SCN^−^ > ClO_3_^−^ > NO_3_^−^ >> Br^−^ > BF_4_^−^ > IO_4_^−^ [25]. NIS can also transport radioactive isotopes used in clinical medicine, especially in diagnostic techniques (^188^ReO_4_^−^, ^18^F-BF_4_^−^, ^211^At, and ^99m^TcO_4_^−^). Later, iodine is transported from the cytoplasm of the epithelial cell to the colloid of the thyroid follicle through the apical membrane. Transport through this membrane involves several molecules whose capacity (or affinity) to transport iodine is much lower than NIS, but they take advantage of the favorable gradient of iodine that NIS has previously created inside the epithelial cell. So far, three transporters have been identified: pendrin, anoctamin 1/TMEM16A, and perhaps cystic fibrosis transmembrane conductance regulator (CFTR) [13]. The participation of these transporters in the process remains to be fully characterized, but mutations in these proteins have been related to thyroid pathologies, especially congenital hypothyroidism. Following the process of the synthesis of thyroid hormones, the enzyme TPO incorporates iodine into the tyrosine residues of the thyroglobulin molecule to give rise to the iodinated residues mono- and di-tyrosine iodine (MIT and DIT, respectively) [26]. This process, also known as organification, involves the oxidation of iodine. For this oxidation, the presence of H_2_O_2_ is required, which is provided by the Dual oxidase 2 (Duox2) molecule [27].

The same enzymes, TPO and Dual oxidase 2 (Duox2), are responsible for coupling two iodinated residues to form the pre-hormones T_3_ and T_4_ within the TG [26]. Iodinated TG (TG-I) is accumulated in the colloid, where it constitutes about 80% of total proteins. Next, and depending on the TH metabolic needs, it can be endocytosed into the cell cytoplasm. TG-I is digested and T_3_/T_4_ are released from TG. The iodine from MIT and DIT residues that did not form TH is also released by the iodotyrosine deiodinase (IYD/DEHAL) enzyme [28] and recycled for new TH synthesis. Finally, T_3_ and T_4_ are transported from the cell cytoplasm to the bloodstream through the Monocarboxylate transporter 8 (MCT8) at the basolateral membrane [29].

Most of the mentioned mechanisms are regulated by the blood concentration of TSH. It binds to its receptor (TSH-R) on the basolateral membrane of the thyroid follicle, and this, in turn, activates different signaling pathways in the cell to regulate the expression, function, degradation, and trafficking of the mentioned molecules (NIS, TPO, TG, Duox2, MCT8), as well as cell proliferation and thyroid growth [18,29,30,31,32]. Furthermore, the production of TSH in the pituitary is inversely regulated by the blood concentration of TH [32]. TG is also usually found in blood, with concentrations between 5 and 25 µg/L being considered normal. These levels can be increased in thyroid hyperplasia, subacute thyroiditis, or Graves’ disease, and in the case of thyroid tumors and/or their metastases [33,34].

Another important regulator in the synthesis of thyroid hormones is iodine itself. Very high blood iodine concentrations lead to iodine self-regulation in the thyroid, also known as the Wolff–Chaikoff effect [35,36,37]. Excess iodine triggers several responses in the thyroid: (i) inhibition of TPO and Duox2, and therefore inhibition of iodine organification in TG, (ii) reduction in expression of NIS mRNA, and (iii) it has recently been shown that it also inhibits the function of NIS at the plasma membrane; this is a very rapid effect [35,38]. The Wolff–Chaikoff effect lasts 2–3 days and is molecularly explained by the excessive oxidation of iodine in different proteins and lipids, which leads to an increase in reactive oxygen species (ROS) [35]. The escape of this effect, or the return to normal thyroid function, occurs after 4–10 days and is due to the action of antioxidant enzymes, mainly thioredoxin reductase 1 (TxnRd1), which reduces ROS levels and allows the re-expression of NIS and TPO [35,38,39]. Recent works have shown using in vitro and in vivo approaches that iodinated contrast agents also affect the incorporation of iodine into the thyroid in an effect that can last for more than eight days, longer than the Wolff–Chaikoff effect [40,41]. These authors reached several important conclusions: (a) that the effect drastically reduces the expression of NIS, which can last more than four days; (b) that it is specific to the thyroid, since it does not affect the expression of NIS in salivary glands; and (c) that it is independent of free iodine. In a proteomics approach, the same group has found different cellular pathways that are modulated both by iodine excess and by iodinated contrast agents [40]. Perhaps the most interesting, communally regulated by both, are the elF4 and P706SK cell signaling pathways and the insulin receptor, since both pathways that have been shown to be involved in the regulation of NIS [42,43,44]. Furthermore, the TSH receptor is also downregulated by iodinated contrast agents.

Finally, there are other factors that regulate iodide uptake and TH synthesis (Table 2). It is noteworthy that most of them are inhibitory and compensate for the powerful stimulating effect that TSH exerts on the thyroid cell.

### 2.2. Thyroid Tumor Cell

In thyroid carcinogenesis of epithelial cell origin, the expression and/or location of some of the described molecules are altered (Table 3 and Figure 2b). It is essential to know what happens to the proteins involved in these processes during tumor development to understand and evaluate possible treatments and to predict clinical results. In differentiated thyroid carcinoma (DTC), both papillary (PTC) and follicular (FTC), many of the properties of normal follicular cells remain. Therefore, although decreased, the expression of TSH-R, NIS, pendrin, TPO and TG is preserved, while the expression of Duox2 is not altered or is slightly increased, and these tumor cells can even synthesize TH [45,46,47,48,49,50]. In some cases, however, these proteins are expressed but may be delocalized and not functional. This has been seen for TPO, Duox2, pendrin and especially for NIS [48,49,51,52,53,54,55]. Many DTC express NIS, even abundantly, but because it is not localized at the plasma membrane, tumor cells are unable to capture iodine [51,56,57].

Hence, the first limiting step is the incorporation/accumulation of iodine radioisotopes and other clinically used isotopes (Table 1). This depends on the amount of NIS present at the plasma membrane of the cell. However, since NIS is capable of incorporating more than 40 times the blood iodine concentration into the cell [23,58], even a small amount of NIS expression would be able to incorporate the necessary radioiodine for the therapy to be effective. Such efficacy will also depend on other factors. One of them is the amount of radioiodine that can be oxidized/organized. Although TPO and TG levels are also decreased in DTC cells, the amount of radioiodine that they can incorporate and organify is high. Another factor is the amount of iodized TG previously stored in the tumor tissue. A low iodine diet will allow for low levels of iodized TG before therapy, and more TG available to be organified by radioiodine. This would consequently increase the effectiveness of the treatment. This has been experimentally demonstrated in vivo in euthyroid mice [59], where it was observed that both a low iodine diet and rhTSH injections in mice treated with T3 significantly increased the accumulation of iodine in the thyroid. Molecularly, they observed that this effect was mainly due to an increase in the expression of NIS. Therefore, in DTC cells where some of the mechanisms that regulate the expression of NIS are still maintained, the phenomenon could be similar.

A decrease in TSH-R in DTC has also been observed in parallel with the decrease in NIS expression (Table 3). Therefore, depending on the amount of TSH-R present in the tumor cell, stimulation with TSH, either endogenous or exogenous, will be more or less effective. In parallel, the expression of TSH/TSH-R dependent proteins will vary, as is the case for NIS, TG, and TPO [45,60]. It has been observed that chronic TSH stimulation, which occurs after the induction of hypothyroidism to raise endogenous TSH, does not alter TSH-R levels and could lead not only to the increased expression of NIS, TG and TPO, but also to an increase in the proliferation and growth of tumor cells [30]. For rhTSH treatment, the situation will be similar, except for the increase in proliferation since the treatment time is very short.

Another important factor that could affect the effectiveness of RAI is the presence of ROS. In DTC, an increase in ROS is observed, mainly as a result of Duox2 (not because the expression of Duox2 increases, but by decreasing the expression of TPO, the production of H_2_O_2_ generated by Duox2 is not consumed and accumulates in the tumor cell) [61]. NOX4 expression is also increased in DTC, leading to an increase in oxidant species that cause DNA damage and promote cell dedifferentiation, tumorigenesis, and chromosomal instability [62]; NOX4 also stimulates TGF-β in cancer [63], which has been shown to play a key role in the BRAF^V600E^-induced repression of NIS [57,64,65].

Massive genomic sequencing has revealed an additional level of complexity in DTC that could help to predict the success of RAI and/or establish new strategies to treat these tumors. The Cancer Genome Atlas (TCGA) Research Network analyzed the genomic landscape of 496 PTCs [66] and established a new molecular classification of PTC. The study suggests that besides harboring the two main drivers of PTC, BRAF and RAS mutations, tumors also have distinct subtypes and signaling pathways that are affected, establishing a more genetically complex scenario than previously thought [66]. In general, BRAF-like tumors have a less differentiated gene expression pattern than RAS-like or other gene fusion tumors. In the case of the genes responsible for iodine uptake and metabolism expression, they are greatly reduced in BRAF-like tumors, in contrast to RAS-like tumors in which the expression of these genes is largely preserved [66,67]. These results (summarized in Table 3) are in agreement with studies in patients which have analyzed thyroid differentiation markers by mRNA [46,47,48,68] or protein [51,53,55,57] analysis.

Thyroid differentiation genes are significantly diminished in poorly differentiated (PDTC) or anaplastic (ATC) thyroid cancers. TG and TSH-R expression are considerably reduced, although they are still detectable (Table 3). In addition, the presence of NIS, TPO and pendrin is practically undetectable, so diagnosis and RAI therapy are usually not effective. The main strategy to treat these tumors is NIS gene (*SLC5A5*) expression re-induction. The current pharmacological drugs used in in vivo or in clinical trials target the Mitogen-Activated Protein Kinase (MAPK) pathway using MAPK inhibitors such as vemurafenib or dabrafenib, tyrosine kinase inhibitors such as sorafenib or cabozantinib, and Mitogen-activated protein kinase kinase (MEK) inhibitors such as selumetinib, refametinib, and PD98059 (for extensive reviews, see: [69,70,71]. Furthermore, other essential thyroid pathways implicated in NIS expression have been targeted. These include, among others: PI3K pathway inhibitors such as LY294002, serine/threonine-protein kinase (Akt) inhibitors such as apigening and rapamycin, and Histone deacetylase (HDAC) inhibitors such as sodium butyrate, trichostatin A, valproic acid, romidepsin, panobinostat, and vorinostat. Other strategies have targeted the Notch1 pathway with inhibitors such as resveratrol or hesperetin. The regulation of NADPH oxidase 4 (NOX4) by the antioxidant alpha-lipoic acid has also been used to enhance radio-iodide accumulation. Most of the above-mentioned pharmacological drugs have shown thyroid tumor cell re-differentiation capacity in in vitro approaches, but clinical results are still on going.

### 2.3. Metastatic Differentiated Thyroid Cancer Cells

DTC metastatic tumor cells, in comparison with primary tumor cells, experience a considerable reduction in the amount of essential genes necessary for RAI, such as TG, TSH-R, and Duox2, but a relatively low reduction of NIS and TPO genes (Table 3) [72]. NIS protein expression in lymph node metastatic DTC tissue has been reported to be around 70%, but only around 40% is localized at the plasma membrane [73]. In general, NIS negative expression is also related to low TSH at the time of tumor resection [74]. Much of the TG synthesized by these cells is discharged into the bloodstream since, in many cases, there is no adequate follicular structure, and therefore iodized or non-iodized TG cannot be stored in the colloid. The determination of high concentrations of TG in the blood after thyroidectomy and RAI therapy is usually an indication of the presence of metastases [75].

The oxidation/organification of iodine in TG or other molecules can be mediated not only by the TG/Duox2 system, but also by other more abundant enzymes in serum, such as LPO and other oxidase enzymes that produce H_2_O_2_, such as the NADPH oxidase (NOX) family [52]. Therefore, the second important step in the success of RAI therapy for metastases will depend on the oxidation of iodide in these cells, which will be mediated by the ability of the cells to store/retain TG-I, or whether cytoplasmic radioiodine can be oxidized to other molecules thanks to the production of H_2_O_2_ by Duox2 and/or NOX enzymes [52].

Most available evidence from patients treated with RAI for metastatic DTC is based on retrospective analysis [76]. Loss of RAI accumulation in distant metastases (DM) is the most significant factor for patient disease-free survival (DFS), independently of whether DM was detected in the initial diagnosis or during follow-up [77]. This can be an effective treatment modality and it contributes significantly to patient life expectancy. Nonetheless, it is necessary to clarify several aspects in the management of this malignancy, such as which activity to use and how to determine this activity, as well as the potential long-term complications [76]. Also, special considerations are necessary in pediatric patients regarding the weight-adaptation of activities, as well as the risk of pulmonary fibrosis in patients with diffuse miliary metastases.

In summary, the expression of the most relevant proteins involved in the accumulation of iodine and its subsequent organification (NIS, TPO, TG, Duox2), as well as its main regulator TSH-R, are reduced or delocalized during thyroid tumor progression. The success of diagnostic imaging techniques and radioiodine therapy depends on the expression levels of these proteins, as well as their correct subcellular location.

## 3. Thyroid Hormone Deprivation vs. Recombinant Human TSH: Pros and Cons

### 3.1. Which TSH Stimulation Treatment Obtains Higher Radioiodide Accumulation and Organification in Tumor Cells?

**Figure 3:**
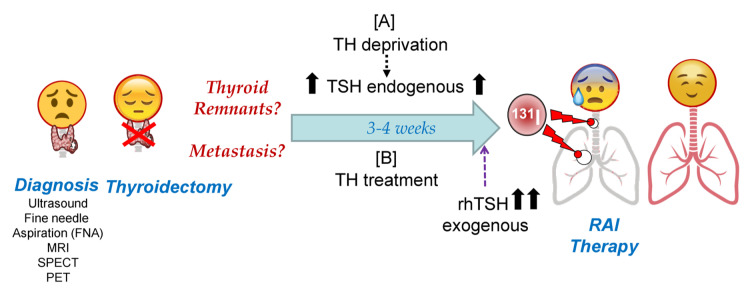
Schematic representation of Radioactive Iodide (RAI) therapy in patients with differentiated thyroid cancer (DTC). After tumor diagnosis, the final goal of both pre-RAI treatments is to achieve maximum radioiodine accumulation in tumor cells, with maximum residence time during ^131^I therapy. Molecularly speaking, this translates to the maximum accumulation of radioiodine through NIS and the maximum iodine organification in TG or other molecules that are able to oxidize iodide. Abbreviations: TH: Thyroid Hormones; TG: thyroglobulin; TSH: Thyroid Stimulating Hormone; rhTSH: recombinant human TSH; MRI: Magnetic resonance imaging; SPECT: Single-photon emission computed tomography; PET: Positron emission tomography.

RAI therapy strategies in DTC are summarized in Figure 3. Given the high capacity of NIS to accumulate iodine against its concentration gradient [58], a very high expression of NIS is not required, but it must be located in the plasma membrane. This could explain, at least in part, why recent studies show that the use of only 30 mCi of ^131^I can have as effective an outcome on remnant ablation as the most common clinically used dose of 100 mCi [78,79]. To achieve maximum iodine organification in TG, and also to keep follicular structures, the presence of the oxidative enzymes TPO and Duox2 is important. Also, in case of TH deprivation, a low iodine diet is very important before radioiodide treatment so that the accumulated/stored TG-I is as low as possible before the therapeutic dose of radioiodide is administered [59].

Both hormonal deprivation and rhTSH can be extrapolated, in part, to in vitro cultures of thyroid cells. In the case of hypothyroidism induced by hormonal deprivation, TSH levels remain chronically high. In in vitro cultures, this chronic TSH stimulation has been shown to result in a continuous synthesis of NIS, TPO, TG, and Duox2, in addition to faster cell growth. However, the chronic induction of these genes results in relatively low expression levels [31]. Additionally, patients need to maintain a significantly low iodine intake because if they did not, part of the expressed TG would be iodized, and subsequent radioiodine organization would be much less effective during therapy. On the other hand, TH deprivation induces high concentrations of TG in colloids and/or in the blood. High TG concentrations result in lower NIS levels [80], and this could affect the efficiency of the organization process during therapy (Table 2). This situation occurs during TH deprivation. Furthermore, the TG in the colloid undergoes different oligomerization processes to allow better storage [26], which can later partially prevent the incorporation of radioiodine into TG, or at least reduce the efficiency of the process.

To prepare for rhTSH treatment, patients are treated with TH to keep endogenous TSH levels very low, and TSH-R protein at low or insignificant levels (Figure 3). Then, there is a high peak of serum TSH with rhTSH treatment. However, the time before the ^131^I treatment (in which TSH levels are high in the patient’s body) is short. In cell culture, the lack of TSH in the medium usually results in the decrease, or even absence, of NIS, both in the plasma membrane and in the cytoplasm [31], and also a considerable reduction in TG expression. Subsequently, the treatment with high TSH produces de novo synthesis of NIS, TG, and TPO, with maximum expression levels after 24–72 h [58]. These expression levels are higher than those obtained with chronic TSH treatment, at least in the case of NIS [31]. Additionally, because the temporal rhTSH stimulus is short, cell growth rate is lower compared to chronic treatment. This could be beneficial for the patient when it comes to tumor cells.

TH treatment inhibits TSH synthesis, and consequently, NIS is not synthesized in the thyroid follicle cell. Therefore, TG will not be iodized before radioiodine treatment and, after TSH stimulation, the efficiency of the organification of radioiodine in TG synthesized de novo will be higher. The duration of the stimulation of rhTSH is shorter than chronic TSH, and therefore a reduction in cell proliferation rate (and, as a consequence, the overall amount of TG protein synthesized), will be lower both in the thyroid cells and in the serum, but proportionally less iodinated.

The amount of radioiodine typically used for ^131^I therapy, regardless of the type of TSH pretreatment, is very high, and only part of it will be transported, accumulated, and organified in normal or tumor thyroid cells. This implies that with large doses of radiation, the relative observed differences in the incorporation and organization in both TSH treatments are not very relevant in terms of the final ablation response. However, there is controversy in the literature regarding dose effects. Different studies compared 30 vs. 100 mCi doses of RAI for post-thyroidectomy low-risk DTC patients, showing similar results in ablation independently of the TSH pre-therapy treatment used [78,79,81,82]. More recent studies did not show similar results and determine that the ablation rate was better with a higher dose of RAI [83]. In intermediate-risk or high-risk DTC patients, low doses appeared inadequate for achieving successful ablation [84]. However, the follow-up period for all these studies was too short (less than one year) to determine whether long-term disease-free survival (DFS) or overall survival (OS) was equivalent.

With respect to differences between TSH stimulation treatments pre-RAI, these tend to be minimal. Clinical data showed that there is a higher, but not significant, initial accumulation of iodine with TH deprivation. On the other hand, some studies showed that rhTSH treatment obtains a significantly greater retention of radioiodine during ^131^I therapy [85,86], probably as a consequence of the greater efficiency of organification, and of a lesser discharge of TG into the bloodstream. This could be observed as a greater amount of radioiodine in whole-body scan images. These differences could become significantly more relevant if the amount of therapeutic radioiodine used is lowered (Table 4).

In summary, TH deprivation keeps serum TSH chronically elevated (Figure 3), leading to the constant expression of NIS and TG in addition to high tumor cell proliferation. Globally, TG expression levels are high, although a significant proportion will be discharged into the bloodstream and part of the TG in the colloid may be organified with cold iodine. This translates into relatively inefficient radioiodine organization during ^131^I treatment.

In recombinant human TSH treatment, endogenous TSH levels are very low or not present, as are NIS and TG expression levels. After treatment with rhTSH, high de novo synthesis of NIS and TG is achieved. This allows for very high NIS radio-iodide uptake and a greater efficiency of radioiodine TG organization in the colloid, increasing radioiodine residence time in thyroid remnants. This approach can compensate for the lower overall expression of TG and prevent the constant proliferation of tumor cells as compared to TH deprivation.

### 3.2. Which TSH Stimulation Treatment Obtains Longer Radioiodine Residence Time in Tumor Cells?

As previously mentioned, in the case of TH deprivation, TG levels are higher in addition to increased proliferation and growth (Table 4). Therefore, the global TG amount will be greater than in thyroid remnants treated with recombinant human TSH. However, part of this TG may be iodized and/or in tertiary structures stored in the colloid, where the incorporation of radioiodine will be less efficient than in the case of de novo synthetized TG, as occurs in the case of stimulation with rhTSH. This would explain why there is a greater amount of organized radioiodine in TH deprivation, and yet the period of time that this radioiodine stays in the remaining thyroid tissue is low [85,86]. Furthermore, chronic TSH will stimulate the proliferation and growth of tumor cells [30]. In the case of TH deprivation, it has been observed that a large part of TG is discharged into the blood before treatment, again decreasing the effectiveness of radioiodine incorporation, and thus allowing a greater exposure of TG to the immune system, which may increase the probability that new anti-TG antibodies are generated. In any case, clinical data indicate that there are no significant differences regarding ablation results obtained in both situations [78,79,85]. This could be explained because the amount of radio-iodide typically used is very high. No significant differences were observed with respect to the recurrence risk in patients treated with either treatment [87,88].

Another important aspect may be kidney function. In TH deprivation, and therefore chronic elevated TSH, kidney function is affected. Since NIS is also expressed in the kidney, normal metabolism iodide flow can be altered [13]. This is important and explains, at least in part, why in TH deprivation the levels of radioiodine in blood, and in general in whole-body scans, are higher than in euthyroid patients treated with rhTSH. However, because kidney function is altered, blood radioiodine levels could increase the risk of side effects in other organs (stomach, ovary, salivary glands, etc.) where NIS is also expressed [13,21]. This would decrease potential radioiodine accumulation in thyroid remnants and/or metastases. In this sense, recent studies demonstrate that DTC patients treated with ^131^I after TSH stimulation have lower abdominal (liver, stomach, ascending colon, transverse colon, descending colon, rectum, and small intestine) radioiodine activity, both in terms of activity ratio and absorbed dose ratios, than patients with TH withdrawal [89]. These results could be relevant to prevent possible gastrointestinal side effects after therapy and also in post-therapy patient management (i.e., length of hospital stay, health cost, and quality of life) [89].

In summary, kinetic data from patients indicate that there are differences in the amount of radioiodine that can accumulate and/or be organified depending on whether the treatment prior to radioiodine is via TH deprivation or by exogenous stimulation with TSH. There is a greater quantity of TG and therefore radioiodine organification in a situation of TH deprivation, but a large part of that TG-I is discharged into the bloodstream, becoming a relatively inefficient process. TH treatment and recombinant human TSH stimulation achieve lower total organification but allow a more efficient accumulation in thyroid remnants. The opposite occurs with radio-iodide residence in blood and other tissues, minimizing possible side effects in other organs (Table 4).

### 3.3. Do Different TSH Stimulation Treatments Affect Negative Scans in Diagnosis?

The management of DTC patients with rising TG levels and negative radioiodine whole body scans is still controversial [90]. A radioiodine negative scan can be mainly due to two factors: (i) NIS expression is very low or null, and (ii) even if there is expression, even over-expression of NIS, the protein is delocalized and does not reach the plasma membrane. Both cases can occur regardless of the type of previous TSH treatment and will depend more on the degree of dedifferentiation of tumor cells and the expression of TSH-R. Iodide negative scans will be more frequent in more de-differentiated cases within the umbrella of those considered DTC. The new classification of DTC from panels of mutated and/or altered genes after sequencing of many patients [66] may help to correlate tumor subtypes more clearly and predict the response to radioiodine treatment. Alternative radioelements, i.e., those not transported by NIS, have been proposed to scan thyroid remnants and/or metastases [91,92,93]. The treatment of those cases may also differ from RAI and strategies used in PDTC and ATC, previously mentioned, could be more promising [69].

### 3.4. What TSH Stimulation Treatment Could Be Better for DTC Metastases?

As in non-metastatic tumor thyroid tissue, the efficacy of radioiodine therapy will depend mainly on the presence of NIS to accumulate iodine and on the TPO/Duox2 system to oxidize radio-iodide into TG radioiodine. However, the organification of iodine can be carried out by other oxidative protein systems such as Lactoperoxidase (LPO)/NOX, and protein targets could be different than TG. This last aspect becomes more important in the treatment of metastasis since higher concentrations of radioiodine are used.

There is still debate regarding the efficacy of the treatment of DTC thyroid metastases when comparing TH deprivation vs. rhTSH [94]. In metastatic patients where radioiodine therapy was used compassionately, both pre-therapy methods were found to be equally effective in dosimetry measurements of patients [94]. Nevertheless, the studies are mostly retrospective, and groups of patients were heterogeneous. In addition, some studies did not find significant differences in the efficacy of eliminating locoregional and pulmonary metastases [95,96], or bone metastases [95,97]. Better programmed epidemiological studies that use a higher number of patients are needed in order to obtain adequate scientific evidence in this regard. One of the most recent studies [98] indicates that radio-iodide retention rate and effective half-time in metastatic lymph nodes were significantly lower than in thyroid remnants of primary tumors. This is concordant to lower levels of expression or delocalization of NIS in metastatic cells (Table 3). Additionally, this study indicates that the retention rate and the effective half-time of thyroid remnants and in metastatic lymph nodes in the rhTSH pre-RAI treatment group were higher than those in the TH deprivation group, although not statistically significant for metastatic cases [98]. Taking into account the clinical response after RAI, overall patient survival after 5.5 years was similar when the pre-RAI treatment was either TH deprivation or rhTSH [97].

In summary, although there are no available large comparative epidemiological studies of RAI treatment of metastases after TH deprivation or rhTSH, most studies show similar effectiveness.

## 4. Stunning Phenomenon in RAI Therapy

Thyroid stunning was first reported in 1951 [99]. This phenomenon is characterized by less ^131^I accumulation at therapy than was predicted from the radioiodine accumulation measurement during dose planning at diagnosis. There is quite a bit of controversy regarding the existence of such an effect, and the possible underlying mechanisms in the stunning process. A wide variety of results both in vitro [100,101,102] and in vivo [103,104,105] have been published, sometimes contradictory; at other times, procedures used differ and results are difficult to compare [106].

Two different effects would explain a large part of the observed results. (1) If radioiodine, either ^131^I, ^123^I, or ^124^I, is used in the initial screening, it will end up oxidized, mostly in TG. This organified radioiodine will be able, depending on quantity and radiation energy levels, to affect the cell where it has been incorporated and even destroy it. Surrounding cells could also be affected through non-targeted reactions (bystander and abscopal effects) [7] as occurs in typical ^131^I therapeutic doses. Therefore, these cells would no longer be able to accumulate or incorporate radioiodine during treatment. Consequently, these cells will have been successfully treated, even with apparently low radioiodine doses, and thyroid stunning would be an artifact due to an early therapeutic effect of ablative ^131^I, and not a clinical problem. In this sense, using ^131^I during diagnosis would be more effective than ^123^I, since ^131^I has higher levels of radiation emission (Table 1). (2) If radioiodine does not destroy the cell, it would be stored as TG-^131^I/^123^I, and this would diminish the future capacity of that TG to organize radioiodine during treatment. In this sense, both radioisotopes would interfere similarly. In these two effects, there would be an additional indirect effect produced by the increase of ROS [107], which would affect the cell depending on accumulated levels. Again, ^131^I would be more effective than ^123^I here. Given that tumor cells already have high levels of ROS, the increased ROS could additionally contribute to the elimination of the cell regardless of radiation. This would be equal in diagnosis and treatment. Using ^123^I and ^124^I may have some disadvantages, such as lower sensitivity, higher costs, shorter half-life, and availability problems [108]. ^124^I has an additional disadvantage because it is a positron emitter which can cause methodological problems.

Other radioisotopes used in diagnosis [100,109] that can be transported by NIS, such as ^99m^TcO_4_, ^211^At, and ^18^F-BF_4_^−^, will not be organified in TG (Table 1). Therefore, the first effect would not occur, and the other two effects would be greatly diminished. As there is no organification in TG and they have short half-lives (Table 1), diagnostic images should be recorded shortly after radioisotope administration. ROS production using theses isotopes, except ^211^At, will be lower than using ^131^I, ^123^I, or ^124^I. Therefore, the isotopes that could cause a minor stunning effect would be those that are not organified by TG, such as ^99m^TcO_4_^−^ and ^18^F-BF_4_. This was demonstrated for ^99m^TcO_4_^−^, where the stunning effect in mice could only be observed at extremely high thyroid absorbed dose thresholds (above 20 Gy), a level unlikely to be found in clinical practice [110]. However, ^99m^TcO_4_^−^ has the drawback of low sensitivity, especially for metastatic images, so a negative diagnosis cannot be absolutely guaranteed [111]. Perhaps the most promising is ^18^F-BF_4_^−^, which provides more sensitive images using positron emission tomography (PET), although more studies are needed to confirm this.

Concerning the first effect, in addition to cellular damage, ROS could generate an effect similar to the Wolff–Chaikoff effect during diagnosis or treatment [35,39,112]. This would cause not only an oxidation of iodide (both TG and lipid oxidation), but also a very rapid inhibition of NIS present at the plasma membrane, even in times as short as 1 h, and could last up to 48–72 h [35,112]. In this case it would be necessary to wait for the escape of the Wolff–Chaikoff effect so that the cells are able to accumulate ^131^I again [35]. Given that ROS are high in tumor cells before diagnostic ^131^I is applied, this phenomenon could be possible even if relatively low doses of ^131^I are used [113].

Clinical results show that using ^131^I at concentrations between 1–3 mCi for diagnosis [114] and applying the therapeutic dose within 24–48 h prevents the stunning effect [106]. However, this effect is observed when using 2–5 mCi of ^123^I for diagnosis. Recent studies have demonstrated the stunning effect in benign thyroid diseases [103], and that it is dependent on pre-therapeutic radiation doses. A correction factor for the therapeutic dose, to avoid the stunning effect, has been proposed [103,115].

One of the controversies in RAI is the most appropriate time-window to administer ^131^I. It has been shown in clinical studies that the greatest accumulation of radioiodine occurs 24–48 h after the injection of rhTSH [116]; therefore, this would be the most appropriate window to use radioiodine for diagnosis and/or treatment. According to this, the closer the doses are given, the better the therapeutic outcome [116].

In summary, based on current data, the best way to avoid stunning would be to use radioisotopes that are not organified in the TG and that are also transported by NIS, such as ^18^F-BF_4_^−^, in diagnostic tests. In clinical studies, it has been shown that an initial dose of 1–3 mCi of ^131^I or 2–5 mCi ^123^I does not generate, or minimally generates, the stunning effect. It is most appropriate to give radioiodine doses prior to 72 h post-injection of recombinant human TSH.

## 5. Imaging Techniques in Thyroid Cancer

RAI therapy imaging techniques in thyroid cancer will depend on the functional expression of NIS in tumor cells, and the availability of the radioisotope (Table 1). Therefore, imaging approaches will be different in DTC than in PDTC or ATC. In DTC, NIS transported photon emitter radionuclides such as ^123/1245/131^I and ^99m^TcO_4_^−^ can be detected by the non-invasive single-photon emission computed tomography (SPECT) imaging technique for medical diagnostic imaging. ^99m^TcO_4_^−^ has a short half-life and a high level of photon emission with appropriate energy for medical diagnostic imaging, but it is not very abundant [117]. ^123^I is also a good radioisotope for SPECT, with a low half-life and relatively close to ^99m^TcO_4_^−^ in photon emission energy, which provides higher sensitivity and imaging quality than ^125^I or ^131^I. ^125^I has low photon energy and an undesirably long half-life, which limits its use in SPECT. ^131^I is used both in diagnostic imaging and therapy. ^131^I emits both beta particles and photons with higher energy than ^99m^TcO_4_^−^ or ^123^I, which provides poorer quality images. In addition, its long half-live increases the unwanted exposure of patients and medical staff.

NIS transported radioisotopes, such as ^124^I and [18F]-tetrafluoroborate (^18^F-BF_4_^-^), can be detected by the highly sensitive non-invasive imaging technique PET. Using ^124^I for PET provides higher imaging sensitivity than SPECT. However, ^124^I emits not only positrons, but also gamma radiation, which disturbs quantitative PET imaging and image quality in several ways [118,119]. Perhaps the most promising NIS transported radioisotope for imaging is ^18^F-BF_4_^−^. It has high positron emission and low energy, which provides 3D PET images with high quality and resolution. It also has the advantage of not being organized in TG.

The use of another NIS-transported isotope, ^211^At, has also been proposed. However, other transporters participate in its incorporation too, and accumulation in the liver, lung, and kidneys has been observed. As a consequence, these non-tumor tissues could be affected by treatment with ^211^At [120].

In undifferentiated tumors, since NIS is not expressed, it is necessary to used radioisotopes other than those carried by NIS for imaging techniques. ^18^F-FDG is transported into the cell via glucose transporters (GLUT) and can be detected by PET. A large variety of tumors overexpress GLUT1 [121], and therefore are capable of differentially accumulating 2-deoxy-2-[18F]-Fluor-D-glucose (^18^F-FDG) compared to non-tumor tissue. However, in the thyroid follicle cell, the expression of GLUT1 is very low, and it only slightly increases in DTC (Table 3). So, ^18^F-FDG accumulation is very low and this makes image acquisition difficult. On the other hand, in more aggressive tumors such as ATC and PDTC, GLUT1 is over-expressed [16,46,122], which enhances ^18^F-FDG accumulation and tumor imaging detection by PET. PET imaging of accumulated ^18^F-FDG could also be necessary in cases of metastatic DTC that turn radioiodine-refractory, given that NIS is not expressed or is not localized at the plasma membrane [92]. In these cases, as observed in the most undifferentiated tumors, a metabolic change is observed that leads to the increased expression of GLUT1.

Some studies have shown accidental ^18^F-FDG accumulation in thyroid cancer patients, and these patients had higher TSH levels than those with no accumulation [123,124]. However, TSH values have been associated with hypothyroidism, probably caused by autoimmune thyroiditis. In vitro studies showed that TSH upregulated GLUT1 mRNA in tumor-free cell line (FRTL-5) [125], but not in FTC (ML-1 and FTC-133) or PTC (TPC-1) cell lines [126,127]. However, since the highest expression of GLUT1 occurs in the most aggressive tumors, and this is where the lowest expression of TSH-R is observed (Table 3), it is unlikely that there is a significant influence of TSH on GLUT expression. This would indicate that regardless of how TSH stimulation pre-treatment is conducted, there is no influence on GLUT1 expression and therefore no significant improvements in PET images using ^18^F-FDG.

In summary, TSH does not regulate GLUT1 expression in thyroid tumor cells. GLUT1 expression is very low in DCT, and therefore the use of ^18^F-FDG for PET imaging does not provide improvements in diagnosis compared to the use of NIS radioiodine incorporation by SPECT or PET. However, in more undifferentiated tumors (PDTC and ATC) or in radioiodine-refractory cases, PET imaging of ^18^F-FDG accumulation could be feasible and/or necessary.

## 6. Socio-Economic Facts

The final aspect to consider in any of the pre-radioiodine therapy treatments have to do with socio-economic conditions. All of the studies carried out show an important benefit in the quality of life of patients treated with recombinant human TSH [78,79,85,128,129,130,131], avoiding mobility problems as a consequence of hypothyroidism induced by chronic TSH after 3–4 weeks of TH deprivation. Even though some studies have shown that hypothyroidism side effects can be reduced by maintaining TH deprivation for only two weeks and using levothyroxine substitutions [128], patients still have problems such as reduced physical function, vitality, mental health, and emotional stability [128].

Regarding economic aspects, treatment with recombinant human TSH allows a reduction in the time of hospital stays, which can result in an important economic and clinical benefit for the health system [132,133,134].

## 7. Conclusions

Eighty years after the first use of RAI therapy for hyperthyroidism [1], it is still the first choice of treatment after thyroidectomy for primary and metastatic DTC tumors. Improvements in patient management and significant advances in imaging techniques have allowed us to refine the use of RAI. The success obtained in the treatment of DTC patients (disease-free and overall survival) has turned RAI into a potential therapeutic tool for other tumors that express NIS, such as breast cancer [12], ovarian cancer [11], and testicular carcinomas [10]. However, there are still issues to improve in patient management, such as pre-surgery treatment method (TH deprivation or rhTSH), the amount of radioiodine used, and short and long-term responses to treatment. Massive sequencing techniques are allowing a more accurate classification of traditional thyroid tumor types. This will allow for improvements in the choice of treatment and in the prediction of patient response to therapy. In turn, it will also help to predict RAI-refractory tumors more accurately and to access alternative treatments without the unnecessary use of RAI, or even better, to allow a re-differentiation treatment of the tumor so that the patient can then be successfully treated with RAI. Even though there are still some challenges to address, this therapy poses greater benefits for patients and relatively minor side effects compared to other therapies. This has made RAI one of the major advances in therapeutic nuclear oncology in the last two decades, and turns NIS into one of the favorite theragnostic tools in targeted therapies [13,14,15,17].

## Figures and Tables

**Table 1:** Radioactive isotopes used in clinical medicine for thyroid cancer. ^18^F-FDG: 2-deoxy-2-[18F]-Fluor-D-glucose; ^18^F-BF4-: [18F]-tetrafluoroborate; EC: electronic capture; γ: gamma radiation (high energy photons); β+: positrons from the nucleus; β-: electrons from the nucleus; IT: isometric transition; PET: positron emission tomography; SPECT: single-photon emission computed tomography; TG: thyroglobulin; GLUT: Glucose Transporter.

Isotope	Detection Technique	Transporter	TG Organification?	Energy Emitted Type	Energy Emitted (KeV)	Half Life	Tissue Penetration/Spatial Resolution
^123^I	SPECT	NIS	Yes	EC/γ	159	13 h	
^124^I	PET	NIS	Yes	β+	510	4.17 d	5.0 mm
^125^I	SPECT	NIS	Yes	EC/γ	27	59.4 d	17 µm
^131^I	SPECT	NIS	Yes	β-/γ	364	8.02 d	0.44–2.4 mm
^211^At	PET	NIS, others	No	EC/α	27 y 6900 (α)	7.2 h	65 µm
^99m^TcO_4_^−^	SPECT	NIS	No	IT/γ	140	6.03 h	
^188^ReO_4_^−^	SPECT	NIS	No	β-/γ	155	17 h	10.8 mm
^18^F-BF_4_^−^	PET	NIS	No	β+	511	110 min	
^18^F-FDG	PET	GLUT	No	β+	511	110 min	4.2 mm

**Table 2:** Iodide uptake and the main regulators of thyroid hormone synthesis.

Stimulators	Inhibitor
TSH Selenium	Iodide (I^−^)
	Thyroglobulin (TG)
	Growth factors and cytokines (IGF1, TGFβ, TNFα, TNFβ, IFNγ, ILα, ILβ and IL-6)
	Reactive oxygen species (ROS)
	Iodinated contrast agents

**Table 3:** Expression level modification of proteins involved in iodine transport and their organization in thyroid cancer compared to normal tissue. ND: not detected; PM: plasma membrane; DTC: differentiated thyroid cancer; mDTC: metastasis of DTC; PDTC: poorly differentiated thyroid cancer; ATC: anaplastic thyroid cancer.

	DTC	mDTC	PDTC and ATC
TSH-R	**↓**	**↓↓↓**	**↓↓↓**
NIS	**↓** = (PM/cytoplasm)	**↓↓** (PM/cytoplasm)	ND
Pendrin	**↓↓**		ND
TG	**↓**	**↓↓↓**	**↓↓↓↓**
TPO	**↓** (PM/cytoplasm)	**↓↓** (PM/cytoplasm)	ND
Duox2	= (PM/cytoplasm)	**↓**	ND
GLUT1	= or slightly **↑**	**↑↑**	**↑↑**

↓: slight decrease, ↓↓: decrease, ↓↓↓: high decrease, ↓↓↓↓: Very high decrease, but still measurable, = unaltered.

**Table 4:** Main benefits and disadvantages in the use of TH deprivation vs. stimulation with recombinant human TSH before RAI therapy.

	Benefit	Disadvantages
TH deprivation for 2–4 weeks	- Chronic synthesis of thyroid proteins (TG, NIS, TPO, and Duox2) - High levels of TG protein within the colloid - Higher amount of TG-^131^I in the remnant after RAI	- Increased tumor cell proliferation - Cold iodine organified TG accumulated within the colloid - Colloid-accumulated TG downregulation of NIS expression - TG or TG-^131^I, before or after RAI treatment, present in the bloodstream - ^131^I organification in TG less efficient - Lower half-life of organified ^131^I within remnant tissue - Hypothyroidism induced by chronic TSH after 3–4 weeks of TH deprivation - Longer hospitalization time
Recombinant human TSH	- De novo NIS and TG protein synthesis - High efficiency in the organification of radioiodine in the nascent TG - Longer radioiodine half-life in the remnant - Less radioiodine biodistribution in other organs - Lower amount of TG in the bloodstream - Less proliferation of tumor cells - Better quality of life for the patient - Shorter patient admission time	- Lower amount of ^131^I organification. - Recombinant hTSH cost

## Data Availability

Not applicable.

## References

[1] Hertz S., Roberts A. (1942). Radioactive Iodine as an Indicator in Thyroid Physiology. V. The Use of Radioactive Iodine in the Differential Diagnosis of Two Types of Graves’ Disease. J. Clin. Investig..

[2] Seidlin S.M., Marinelli L.D., Oshry E. (1946). Radioactive Iodine Therapy; Effect on Functioning Metastases of Adenocarcinoma of the Thyroid. J. Am. Med. Assoc..

[3] Gronich N., Lavi I., Rennert G., Saliba W. (2020). Cancer Risk after Radioactive Iodine Treatment for Hyperthyroidism: A Cohort Study. Thyroid.

[4] Lomax M.E., Folkes L.K., O’Neill P. (2013). Biological consequences of radiation-induced DNA damage: Relevance to radiotherapy. Clin. Oncol..

[5] Zhao L.M., Pang A.X. (2017). Iodine-131 treatment of thyroid cancer cells leads to suppression of cell proliferation followed by induction of cell apoptosis and cell cycle arrest by regulation of B-cell translocation gene 2-mediated JNK/NF-kappaB pathways. Braz. J. Med Biol. Res. Rev. Bras. Pesqui. Med. Biol..

[6] Lyckesvard M.N., Kapoor N., Ingeson-Carlsson C., Carlsson T., Karlsson J.O., Postgard P., Himmelman J., Forssell-Aronsson E., Hammarsten O., Nilsson M. (2016). Linking loss of sodium-iodide symporter expression to DNA damage. Exp. Cell Res..

[7] Pouget J.P., Georgakilas A.G., Ravanat J.L. (2018). Targeted and Off-Target (Bystander and Abscopal) Effects of Radiation Therapy: Redox Mechanisms and Risk/Benefit Analysis. Antioxid. Redox. Signal..

[8] Ylli D., Van Nostrand D., Wartofsky L. (2019). Conventional Radioiodine Therapy for Differentiated Thyroid Cancer. Endocrinol. Metab. Clin. N. Am..

[9] Tuttle R.M., Ahuja S., Avram A.M., Bernet V.J., Bourguet P., Daniels G.H., Dillehay G., Draganescu C., Flux G., Fuhrer D. (2019). Controversies, Consensus, and Collaboration in the Use of ^131^I Therapy in Differentiated Thyroid Cancer: A Joint Statement from the American Thyroid Association, the European Association of Nuclear Medicine, the Society of Nuclear Medicine and Molecular Imaging, and the European Thyroid Association. Thyroid.

[10] Micali S., Maggisano V., Cesinaro A., Celano M., Territo A., Reggiani Bonetti L., Sponziello M., Migaldi M., Navarra M., Bianchi G. (2013). Sodium/iodide symporter is expressed in the majority of seminomas and embryonal testicular carcinomas. J. Endocrinol..

[11] Riesco-Eizaguirre G., Leoni S.G., Mendiola M., Estevez-Cebrero M.A., Gallego M.I., Redondo A., Hardisson D., Santisteban P., De la Vieja A. (2014). NIS mediates iodide uptake in the female reproductive tract and is a poor prognostic factor in ovarian cancer. J. Clin. Endocrinol. Metab..

[12] Tazebay U.H., Wapnir I.L., Levy O., Dohan O., Zuckier L.S., Zhao Q.H., Deng H.F., Amenta P.S., Fineberg S., Pestell R.G. (2000). The mammary gland iodide transporter is expressed during lactation and in breast cancer. Nat. Med..

[13] De la Vieja A., Santisteban P. (2018). Role of iodide metabolism in physiology and cancer. Endocr. Relat. Cancer.

[14] Ravera S., Reyna-Neyra A., Ferrandino G., Amzel L.M., Carrasco N. (2017). The Sodium/Iodide Symporter (NIS): Molecular Physiology and Preclinical and Clinical Applications. Annu. Rev. Physiol..

[15] Spitzweg C., Morris J.C. (2004). Gene therapy for thyroid cancer: Current status and future prospects. Thyroid.

[16] Chung J.K., Cheon G.J. (2014). Radioiodine therapy in differentiated thyroid cancer: The first targeted therapy in oncology. Endocrinol. Metab..

[17] Ahn B.C. (2012). Sodium iodide symporter for nuclear molecular imaging and gene therapy: From bedside to bench and back. Theranostics.

[18] De La Vieja A., Dohan O., Levy O., Carrasco N. (2000). Molecular analysis of the sodium/iodide symporter: Impact on thyroid and extrathyroid pathophysiology. Physiol. Rev..

[19] Rousset B., Dupuy C., Miot F., Dumont J., Feingold K.R., Anawalt B., Boyce A., Chrousos G., Dungan K., Grossman A., Hershman J.M., Kaltsas G., Koch C., Kopp P. (2000). Chapter 2 Thyroid Hormone Synthesis And Secretion. Endotext.

[20] Jiang H., DeGrado T.R. (2018). [^18^F]Tetrafluoroborate ([^18^F]TFB) and its analogs for PET imaging of the sodium/iodide symporter. Theranostics.

[21] Portulano C., Paroder-Belenitsky M., Carrasco N. (2014). The Na^+^/I^−^ symporter (NIS): Mechanism and medical impact. Endocr. Rev..

[22] Riesco-Eizaguirre G., Santisteban P. (2007). New insights in thyroid follicular cell biology and its impact in thyroid cancer therapy. Endocr. Relat. Cancer.

[23] Dai G., Levy O., Carrasco N. (1996). Cloning and characterization of the thyroid iodide transporter. Nature.

[24] Levy O., De la Vieja A., Ginter C.S., Riedel C., Dai G., Carrasco N. (1998). N-linked glycosylation of the thyroid Na^+^/I^−^ symporter (NIS). Implications for its secondary structure model. J. Biol. Chem..

[25] Paroder-Belenitsky M., Maestas M.J., Dohan O., Nicola J.P., Reyna-Neyra A., Follenzi A., Dadachova E., Eskandari S., Amzel L.M., Carrasco N. (2011). Mechanism of anion selectivity and stoichiometry of the Na^+^/I^−^ symporter (NIS). Proc. Natl. Acad. Sci. USA.

[26] Di Jeso B., Arvan P. (2016). Thyroglobulin From Molecular and Cellular Biology to Clinical Endocrinology. Endocr. Rev..

[27] De Deken X., Wang D., Many M.C., Costagliola S., Libert F., Vassart G., Dumont J.E., Miot F. (2000). Cloning of two human thyroid cDNAs encoding new members of the NADPH oxidase family. J. Biol. Chem..

[28] Moreno J.C., Visser T.J. (2010). Genetics and phenomics of hypothyroidism and goiter due to iodotyrosine deiodinase (DEHAL1) gene mutations. Mol. Cell Endocrinol..

[29] Visser W.E., Friesema E.C., Visser T.J. (2011). Minireview: Thyroid hormone transporters: The knowns and the unknowns. Mol. Endocrinol..

[30] Garcia-Jimenez C., Santisteban P. (2007). TSH signalling and cancer. Arq. Bras. Endocrinol. Metabol..

[31] Riedel C., Levy O., Carrasco N. (2001). Post-transcriptional regulation of the sodium/iodide symporter by thyrotropin. J. Biol. Chem..

[32] Ortiga-Carvalho T.M., Chiamolera M.I., Pazos-Moura C.C., Wondisford F.E. (2016). Hypothalamus-Pituitary-Thyroid Axis. Compr. Physiol..

[33] Biondi B., Bartalena L., Cooper D.S., Hegedus L., Laurberg P., Kahaly G.J. (2015). The 2015 European Thyroid Association Guidelines on Diagnosis and Treatment of Endogenous Subclinical Hyperthyroidism. Eur. Thyroid J..

[34] Medici M., Visser T.J., Peeters R.P. (2017). Genetics of thyroid function. Best Pract. Res. Clin. Endocrinol. Metab..

[35] Leoni S.G., Kimura E.T., Santisteban P., De la Vieja A. (2011). Regulation of thyroid oxidative state by thioredoxin reductase has a crucial role in thyroid responses to iodide excess. Mol. Endocrinol..

[36] Wolff J., Chaikoff I.L. (1948). Plasma inorganic iodide as a homeostatic regulator of thyroid function. J. Biol. Chem..

[37] Leung A.M., Braverman L.E. (2014). Consequences of excess iodine. Nat. Rev. Endocrinol..

[38] Eng P.H., Cardona G.R., Fang S.L., Previti M., Alex S., Carrasco N., Chin W.W., Braverman L.E. (1999). Escape from the acute Wolff-Chaikoff effect is associated with a decrease in thyroid sodium/iodide symporter messenger ribonucleic acid and protein. Endocrinology.

[39] Leoni S.G., Sastre-Perona A., De la Vieja A., Santisteban P. (2016). Selenium Increases Thyroid-Stimulating Hormone-Induced Sodium/Iodide Symporter Expression Through Thioredoxin/Apurinic/Apyrimidinic Endonuclease 1-Dependent Regulation of Paired Box 8 Binding Activity. Antioxid. Redox. Signal..

[40] Hichri M., Vassaux G., Guigonis J.M., Juhel T., Graslin F., Guglielmi J., Pourcher T., Cambien B. (2020). Proteomic Analysis of Iodinated Contrast Agent-Induced Perturbation of Thyroid Iodide Uptake. J. Clin. Med..

[41] Vassaux G., Zwarthoed C., Signetti L., Guglielmi J., Compin C., Guigonis J.M., Juhel T., Humbert O., Benisvy D., Pourcher T. (2018). Iodinated Contrast Agents Perturb Iodide Uptake by the Thyroid Independently of Free Iodide. J. Nucl. Med..

[42] Garcia B., Santisteban P. (2002). PI3K is involved in the IGF-I inhibition of TSH-induced sodium/iodide symporter gene expression. Mol. Endocrinol..

[43] Kogai T., Sajid-Crockett S., Newmarch L.S., Liu Y.Y., Brent G.A. (2008). Phosphoinositide-3-kinase inhibition induces sodium/iodide symporter expression in rat thyroid cells and human papillary thyroid cancer cells. J. Endocrinol..

[44] Zaballos M.A., Garcia B., Santisteban P. (2008). Gbetagamma dimers released in response to thyrotropin activate phosphoinositide 3-kinase and regulate gene expression in thyroid cells. Mol. Endocrinol..

[45] Lacroix L., Nocera M., Mian C., Caillou B., Virion A., Dupuy C., Filetti S., Bidart J.M., Schlumberger M. (2001). Expression of nicotinamide adenine dinucleotide phosphate oxidase flavoprotein DUOX genes and proteins in human papillary and follicular thyroid carcinomas. Thyroid.

[46] Lazar V., Bidart J.M., Caillou B., Mahe C., Lacroix L., Filetti S., Schlumberger M. (1999). Expression of the Na^+^/I^−^ symporter gene in human thyroid tumors: A comparison study with other thyroid-specific genes. J. Clin. Endocrinol. Metab..

[47] Makhlouf A.M., Chitikova Z., Pusztaszeri M., Berczy M., Delucinge-Vivier C., Triponez F., Meyer P., Philippe J., Dibner C. (2016). Identification of CHEK1, SLC26A4, c-KIT, TPO and TG as new biomarkers for human follicular thyroid carcinoma. Oncotarget.

[48] Bastos A.U., Oler G., Nozima B.H., Moyses R.A., Cerutti J.M. (2015). BRAF V600E and decreased NIS and TPO expression are associated with aggressiveness of a subgroup of papillary thyroid microcarcinoma. Eur. J. Endocrinol..

[49] Bidart J.M., Mian C., Lazar V., Russo D., Filetti S., Caillou B., Schlumberger M. (2000). Expression of pendrin and the Pendred syndrome (PDS) gene in human thyroid tissues. J. Clin. Endocrinol. Metab..

[50] Kondo T., Nakamura N., Suzuki K., Murata S., Muramatsu A., Kawaoi A., Katoh R. (2003). Expression of human pendrin in diseased thyroids. J. Histochem. Cytochem. Off. J. Histochem. Soc..

[51] Dohan O., Baloch Z., Banrevi Z., Livolsi V., Carrasco N. (2001). Rapid communication: Predominant intracellular overexpression of the Na^+^/I^−^ symporter (NIS) in a large sampling of thyroid cancer cases. J. Clin. Endocrinol. Metab..

[52] Carvalho D.P., Dupuy C. (2013). Role of the NADPH Oxidases DUOX and NOX4 in Thyroid Oxidative Stress. Eur. Thyroid J..

[53] Caballero Y., Lopez-Tomassetti E.M., Favre J., Santana J.R., Cabrera J.J., Hernandez J.R. (2015). The value of thyroperoxidase as a prognostic factor for differentiated thyroid cancer—A long-term follow-up study. Thyroid Res..

[54] Faggiano A., Caillou B., Lacroix L., Talbot M., Filetti S., Bidart J.M., Schlumberger M. (2007). Functional characterization of human thyroid tissue with immunohistochemistry. Thyroid.

[55] Skubis-Zegadlo J., Nikodemska A., Przytula E., Mikula M., Bardadin K., Ostrowski J., Wenzel B.E., Czarnocka B. (2005). Expression of pendrin in benign and malignant human thyroid tissues. Br. J. Cancer.

[56] Tavares C., Coelho M.J., Eloy C., Melo M., da Rocha A.G., Pestana A., Batista R., Ferreira L.B., Rios E., Selmi-Ruby S. (2018). NIS expression in thyroid tumors, relation with prognosis clinicopathological and molecular features. Endocr. Connect..

[57] Riesco-Eizaguirre G., Rodriguez I., De la Vieja A., Costamagna E., Carrasco N., Nistal M., Santisteban P. (2009). The BRAFV600E oncogene induces transforming growth factor beta secretion leading to sodium iodide symporter repression and increased malignancy in thyroid cancer. Cancer Res..

[58] Dohan O., De la Vieja A., Paroder V., Riedel C., Artani M., Reed M., Ginter C.S., Carrasco N. (2003). The sodium/iodide Symporter (NIS): Characterization, regulation, and medical significance. Endocr. Rev..

[59] Zwarthoed C., Chatti K., Guglielmi J., Hichri M., Compin C., Darcourt J., Vassaux G., Benisvy D., Pourcher T., Cambien B. (2016). Single-Photon Emission Computed Tomography for Preclinical Assessment of Thyroid Radioiodide Uptake Following Various Combinations of Preparative Measures. Thyroid.

[60] Bruno R., Ferretti E., Tosi E., Arturi F., Giannasio P., Mattei T., Scipioni A., Presta I., Morisi R., Gulino A. (2005). Modulation of thyroid-specific gene expression in normal and nodular human thyroid tissues from adults: An in vivo effect of thyrotropin. J. Clin. Endocrinol. Metab..

[61] Ohye H., Sugawara M. (2010). Dual oxidase, hydrogen peroxide and thyroid diseases. Exp. Biol. Med..

[62] Ameziane El Hassani R., Buffet C., Leboulleux S., Dupuy C. (2019). Oxidative stress in thyroid carcinomas: Biological and clinical significance. Endocr. Relat. Cancer.

[63] Azouzi N., Cailloux J., Cazarin J.M., Knauf J.A., Cracchiolo J., Al Ghuzlan A., Hartl D., Polak M., Carre A., El Mzibri M. (2017). NADPH Oxidase NOX4 Is a Critical Mediator of BRAF(V600E)-Induced Downregulation of the Sodium/Iodide Symporter in Papillary Thyroid Carcinomas. Antioxid. Redox. Signal..

[64] Riesco-Eizaguirre G., Gutierrez-Martinez P., Garcia-Cabezas M.A., Nistal M., Santisteban P. (2006). The oncogene BRAF V600E is associated with a high risk of recurrence and less differentiated papillary thyroid carcinoma due to the impairment of Na^+^/I^−^ targeting to the membrane. Endocr. Relat. Cancer.

[65] Ho A.L., Grewal R.K., Leboeuf R., Sherman E.J., Pfister D.G., Deandreis D., Pentlow K.S., Zanzonico P.B., Haque S., Gavane S. (2013). Selumetinib-enhanced radioiodine uptake in advanced thyroid cancer. N. Engl. J. Med..

[66] The Cancer Genome Atlas Research Network (2014). Integrated genomic characterization of papillary thyroid carcinoma. Cell.

[67] Song Y.S., Park Y.J. (2018). Expression of Sodium-Iodide Symporter Depending on Mutational Status and Lymphocytic Thyroiditis in Papillary Thyroid Carcinoma. Int. J. Thyroidol..

[68] Li J., Dong J.N., Zhao Z., Lv Q., Yun B., Liu J.Q., Cai X.Y. (2018). Expression of sodium/iodide transporters and thyroid stimulating hormone receptors in thyroid cancer patients and its correlation with iodine nutrition status and pathology. Eur. Rev. Med. Pharmacol. Sci..

[69] Buffet C., Wassermann J., Hecht F., Leenhardt L., Dupuy C., Groussin L., Lussey-Lepoutre C. (2020). Redifferentiation of radioiodine-refractory thyroid cancers. Endocr. Relat. Cancer.

[70] Liu J., Liu Y., Lin Y., Liang J. (2019). Radioactive Iodine-Refractory Differentiated Thyroid Cancer and Redifferentiation Therapy. Endocrinol. Metab..

[71] Ljubas J., Ovesen T., Rusan M. (2019). A Systematic Review of Phase II Targeted Therapy Clinical Trials in Anaplastic Thyroid Cancer. Cancers.

[72] Kuo C.Y., Liu T.P., Yang P.S., Cheng S.P. (2017). Characteristics of lymphocyte-infiltrating papillary thyroid cancer. J. Cancer Res. Pract..

[73] Arturi F., Russo D., Giuffrida D., Schlumberger M., Filetti S. (2000). Sodium-iodide symporter (NIS) gene expression in lymph-node metastases of papillary thyroid carcinomas. Eur. J. Endocrinol..

[74] Castro M.R., Bergert E.R., Goellner J.R., Hay I.D., Morris J.C. (2001). Immunohistochemical analysis of sodium iodide symporter expression in metastatic differentiated thyroid cancer: Correlation with radioiodine uptake. J. Clin. Endocrinol. Metab..

[75] Kim H., Kim Y.N., Kim H.I., Park S.Y., Choe J.H., Kim J.H., Kim J.S., Chung J.H., Kim T.H., Kim S.W. (2017). Preoperative serum thyroglobulin predicts initial distant metastasis in patients with differentiated thyroid cancer. Sci. Rep..

[76] Verburg F.A., Hanscheid H., Luster M. (2017). Radioactive iodine (RAI) therapy for metastatic differentiated thyroid cancer. Best Pract. Res. Clin. Endocrinol. Metab..

[77] Albano D., Panarotto M.B., Durmo R., Rodella C., Bertagna F., Giubbini R. (2019). Clinical and prognostic role of detection timing of distant metastases in patients with differentiated thyroid cancer. Endocrine.

[78] Mallick U., Harmer C., Yap B., Wadsley J., Clarke S., Moss L., Nicol A., Clark P.M., Farnell K., McCready R. (2012). Ablation with low-dose radioiodine and thyrotropin alfa in thyroid cancer. N. Engl. J. Med..

[79] Schlumberger M., Catargi B., Borget I., Deandreis D., Zerdoud S., Bridji B., Bardet S., Leenhardt L., Bastie D., Schvartz C. (2012). Strategies of radioiodine ablation in patients with low-risk thyroid cancer. N. Engl. J. Med..

[80] Sellitti D.F., Suzuki K. (2014). Intrinsic regulation of thyroid function by thyroglobulin. Thyroid.

[81] Bal C., Chandra P., Kumar A., Dwivedi S. (2012). A randomized equivalence trial to determine the optimum dose of iodine-131 for remnant ablation in differentiated thyroid cancer. Nucl. Med. Commun..

[82] Prior-Sanchez I., Muñoz-Jimenez C., Moreno-Moreno P., Rebollo-Roman A., Barrera-Martín A., Moreno-Ortega E., Vallejo-Casas J.A., Galvez-Moreno M.A. Our experience with low doses of radioactive iodine (30 mCi) in patients with differentiated thyroid cancer. Proceedings of the 18th European Congress of Endocrinology.

[83] Albano D., Bonacina M., Durmo R., Bertagna F., Giubbini R. (2020). Efficacy of low radioiodine activity versus intermediate-high activity in the ablation of low-risk differentiated thyroid cancer. Endocrine.

[84] Abe K., Ishizaki U., Ono T., Horiuchi K., Kanaya K., Sakai S., Okamoto T. (2020). Low-dose radioiodine therapy for patients with intermediate- to high-risk differentiated thyroid cancer. Ann. Nucl. Med..

[85] Hanscheid H., Lassmann M., Luster M., Thomas S.R., Pacini F., Ceccarelli C., Ladenson P.W., Wahl R.L., Schlumberger M., Ricard M. (2006). Iodine biokinetics and dosimetry in radioiodine therapy of thyroid cancer: Procedures and results of a prospective international controlled study of ablation after rhTSH or hormone withdrawal. J. Nucl. Med..

[86] Taieb D., Sebag F., Farman-Ara B., Portal T., Baumstarck-Barrau K., Fortanier C., Bourrelly M., Mancini J., De Micco C., Auquier P. (2010). Iodine biokinetics and radioiodine exposure after recombinant human thyrotropin-assisted remnant ablation in comparison with thyroid hormone withdrawal. J. Clin. Endocrinol. Metab..

[87] Elisei R., Schlumberger M., Driedger A., Reiners C., Kloos R.T., Sherman S.I., Haugen B., Corone C., Molinaro E., Grasso L. (2009). Follow-up of low-risk differentiated thyroid cancer patients who underwent radioiodine ablation of postsurgical thyroid remnants after either recombinant human thyrotropin or thyroid hormone withdrawal. J. Clin. Endocrinol. Metab..

[88] Tuttle R.M., Brokhin M., Omry G., Martorella A.J., Larson S.M., Grewal R.K., Fleisher M., Robbins R.J. (2008). Recombinant human TSH-assisted radioactive iodine remnant ablation achieves short-term clinical recurrence rates similar to those of traditional thyroid hormone withdrawal. J. Nucl. Med..

[89] Campenni A., Amato E., Laudicella R., Alibrandi A., Cardile D., Pignata S.A., Trimarchi F., Ruggeri R.M., Auditore L., Baldari S. (2019). Recombinant human thyrotropin (rhTSH) versus Levo-thyroxine withdrawal in radioiodine therapy of differentiated thyroid cancer patients: Differences in abdominal absorbed dose. Endocrine.

[90] Chao M. (2010). Management of differentiated thyroid cancer with rising thyroglobulin and negative diagnostic radioiodine whole body scan. Clin. Oncol..

[91] Elboga U., Karaoglan H., Sahin E., Kalender E., Demir H.D., Basibuyuk M., Zeki Celen Y., Yilmaz M., Ozkaya M. (2015). F-18 FDG PET/CT imaging in the diagnostic work-up of thyroid cancer patients with high serum thyroglobulin, negative I-131 whole body scan and suppressed thyrotropin: 8-year experience. Eur. Rev. Med Pharmacol. Sci..

[92] Riesco-Eizaguirre G., Galofre J.C., Grande E., Zafon Llopis C., Ramon y Cajal Asensio T., Navarro Gonzalez E., Jimenez-Fonseca P., Santamaria Sandi J., Gomez Saez J.M., Capdevila J. (2016). Spanish consensus for the management of patients with advanced radioactive iodine refractory differentiated thyroid cancer. Endocrinol. Nutr..

[93] Zakani A., Saghari M., Eftekhari M., Fard-Esfahani A., Fallahi B., Esmaili J., Assadi M. (2011). Evaluation of radioiodine therapy in differentiated thyroid cancer subjects with elevated serum thyroglobulin and negative whole body scan using 131I with emphasize on the thallium scintigraphy in these subgroups. Eur. Rev. Med Pharmacol. Sci..

[94] Klubo-Gwiezdzinska J., Burman K.D., Van Nostrand D., Mete M., Jonklaas J., Wartofsky L. (2013). Potential use of recombinant human thyrotropin in the treatment of distant metastases in patients with differentiated thyroid cancer. Endocr. Pract..

[95] Liepe K. (2015). Sensitivity of preparation with rhTSH or thyroid hormone withdrawal using ^131^I-whole body scans to identify metastases of differentiated thyroid cancer. Int. J. Surg..

[96] Tuttle R.M., Lopez N., Leboeuf R., Minkowitz S.M., Grewal R., Brokhin M., Omry G., Larson S. (2010). Radioactive iodine administered for thyroid remnant ablation following recombinant human thyroid stimulating hormone preparation also has an important adjuvant therapy function. Thyroid.

[97] Tala H., Robbins R., Fagin J.A., Larson S.M., Tuttle R.M. (2011). Five-year survival is similar in thyroid cancer patients with distant metastases prepared for radioactive iodine therapy with either thyroid hormone withdrawal or recombinant human TSH. J. Clin. Endocrinol. Metab..

[98] Hong C.M., Kim C.Y., Son S.H., Jung J.H., Lee C.H., Jeong J.H., Jeong S.Y., Lee S.W., Lee J., Ahn B.C. (2017). I-131 biokinetics of remnant normal thyroid tissue and residual thyroid cancer in patients with differentiated thyroid cancer: Comparison between recombinant human TSH administration and thyroid hormone withdrawal. Ann. Nucl. Med..

[99] Rawson R.W., Rall J.E., Peacock W. (1951). Limitations in the treatment of cancer of the thyroid with radioactive iodine. Trans. Assoc. Am. Physicians.

[100] Lundh C., Lindencrona U., Postgard P., Carlsson T., Nilsson M., Forssell-Aronsson E. (2009). Radiation-induced thyroid stunning: Differential effects of ^123^I, ^131^I, ^99m^Tc, and ^211^At on iodide transport and NIS mRNA expression in cultured thyroid cells. J. Nucl. Med..

[101] Meller B., Gaspar E., Deisting W., Czarnocka B., Baehre M., Wenzel B.E. (2008). Decreased radioiodine uptake of FRTL-5 cells after ^131^I incubation in vitro: Molecular biological investigations indicate a cell cycle-dependent pathway. Eur. J. Nucl. Med. Mol. Imaging.

[102] Postgard P., Himmelman J., Lindencrona U., Bhogal N., Wiberg D., Berg G., Jansson S., Nystrom E., Forssell-Aronsson E., Nilsson M. (2002). Stunning of iodide transport by ^131^I irradiation in cultured thyroid epithelial cells. J. Nucl. Med..

[103] Happel C., Kranert W.T., Ackermann H., Binse I., Bockisch B., Groner D., Herrmann K., Grunwald F. (2019). Thyroid stunning in radioiodine-131 therapy of benign thyroid diseases. Endocrine.

[104] Lassmann M., Luster M., Hanscheid H., Reiners C. (2004). Impact of 131I diagnostic activities on the biokinetics of thyroid remnants. J. Nucl. Med..

[105] Morris L.F., Waxman A.D., Braunstein G.D. (2003). Thyroid stunning. Thyroid.

[106] McDougall I.R., Iagaru A. (2011). Thyroid stunning: Fact or fiction?. Semin. Nucl. Med..

[107] Vrndic O.B., Radivojevic S.D., Jovanovic M.D., Djukic S.M., Teodorovic L.C., Simonovic S.T. (2014). Oxidative stress in patients with differentiated thyroid cancer: Early effects of radioiodine therapy. Indian J. Biochem. Biophys..

[108] Ruhlmann M., Sonnenschein W., Nagarajah J., Binse I., Herrmann K., Jentzen W. (2018). Pretherapeutic ^124^I dosimetry reliably predicts intratherapeutic blood kinetics of ^131^I in patients with differentiated thyroid carcinoma receiving high therapeutic activities. Nucl. Med. Commun..

[109] Watanabe K., Igarashi T., Ashida H., Ogiwara S., Ohta T., Uchiyama M., Ojiri H. (2019). Diagnostic value of ultrasonography and TI-201/Tc-99m dual scintigraphy in differentiating between benign and malignant thyroid nodules. Endocrine.

[110] Cambien B., Franken P.R., Lamit A., Mauxion T., Richard-Fiardo P., Guglielmi J., Crescence L., Mari B., Pourcher T., Darcourt J. (2014). ^99m^TcO_4_^−^-, auger-mediated thyroid stunning: Dosimetric requirements and associated molecular events. PLoS ONE.

[111] Kueh S.S., Roach P.J., Schembri G.P. (2010). Role of Tc-99m pertechnetate for remnant scintigraphy post-thyroidectomy. Clin. Nucl. Med..

[112] Arriagada A.A., Albornoz E., Opazo M.C., Becerra A., Vidal G., Fardella C., Michea L., Carrasco N., Simon F., Elorza A.A. (2015). Excess iodide induces an acute inhibition of the sodium/iodide symporter in thyroid male rat cells by increasing reactive oxygen species. Endocrinology.

[113] Liou G.Y., Storz P. (2010). Reactive oxygen species in cancer. Free Radic. Res..

[114] Yap B.K., Murby B. (2014). No adverse affect in clinical outcome using low preablation diagnostic ^131^I activity in differentiated thyroid cancer: Refuting thyroid-stunning effect. J. Clin. Endocrinol. Metab..

[115] Happel C., Kranert W.T., Groner D., Bockisch B., Sabet A., Vardarli I., Gorges R., Herrmann K., Grunwald F. (2020). Correction for hyperfunctioning radiation-induced stunning (CHRIS) in benign thyroid diseases. Endocrine.

[116] Fast S., Nielsen V.E., Grupe P., Bonnema S.J., Hegedus L. (2009). Optimizing ^131^I uptake after rhTSH stimulation in patients with nontoxic multinodular goiter: Evidence from a prospective, randomized, double-blind study. J. Nucl. Med..

[117] Filzen L.M., Ellingson L.R., Paulsen A.M., Hung J.C. (2017). Potential Ways to Address Shortage Situations of ^99^Mo/^99m^Tc. J. Nucl. Med. Technol..

[118] Lubberink M., Herzog H. (2011). Quantitative imaging of ^124^I and ^86^Y with PET. Eur. J. Nucl. Med. Mol. Imaging.

[119] Pentlow K.S., Graham M.C., Lambrecht R.M., Daghighian F., Bacharach S.L., Bendriem B., Finn R.D., Jordan K., Kalaigian H., Karp J.S. (1996). Quantitative imaging of iodine-124 with PET. J. Nucl. Med..

[120] Spetz J., Rudqvist N., Forssell-Aronsson E. (2013). Biodistribution and dosimetry of free ^211^At, ^125^I^−^ and ^131^I^−^ in rats. Cancer Biother. Radiopharm..

[121] Wang J., Ye C., Chen C., Xiong H., Xie B., Zhou J., Chen Y., Zheng S., Wang L. (2017). Glucose transporter GLUT1 expression and clinical outcome in solid tumors: A systematic review and meta-analysis. Oncotarget.

[122] Kim S., Chung J.K., Min H.S., Kang J.H., Park D.J., Jeong J.M., Lee D.S., Park S.H., Cho B.Y., Lee S. (2014). Expression patterns of glucose transporter-1 gene and thyroid specific genes in human papillary thyroid carcinoma. Nucl. Med. Mol. Imaging.

[123] Bakhshayesh Karam M., Doroudinia A., Joukar F., Nadi K., Dorudinia A., Mehrian P., Yousefikoma A. (2017). Hypermetabolic Thyroid Incidentaloma on Positron Emission Tomography: Review of Laboratory, Radiologic, and Pathologic Characteristics. J. Thyroid Res..

[124] Pruthi A., Choudhury P.S., Gupta M., Taywade S. (2015). Does the intensity of diffuse thyroid gland uptake on F-18 fluorodeoxyglucose positron emission tomography/computed tomography scan predict the severity of hypothyroidism? Correlation between maximal standardized uptake value and serum thyroid stimulating hormone levels. Indian J. Nucl. Med..

[125] Hosaka Y., Tawata M., Kurihara A., Ohtaka M., Endo T., Onaya T. (1992). The regulation of two distinct glucose transporter (GLUT1 and GLUT4) gene expressions in cultured rat thyroid cells by thyrotropin. Endocrinology.

[126] Prante O., Maschauer S., Fremont V., Reinfelder J., Stoehr R., Szkudlinski M., Weintraub B., Hartmann A., Kuwert T. (2009). Regulation of uptake of 18F-FDG by a follicular human thyroid cancer cell line with mutation-activated K-ras. J. Nucl. Med..

[127] Matsuzu K., Segade F., Wong M., Clark O.H., Perrier N.D., Bowden D.W. (2005). Glucose transporters in the thyroid. Thyroid.

[128] Pacini F., Ladenson P.W., Schlumberger M., Driedger A., Luster M., Kloos R.T., Sherman S., Haugen B., Corone C., Molinaro E. (2006). Radioiodine ablation of thyroid remnants after preparation with recombinant human thyrotropin in differentiated thyroid carcinoma: Results of an international, randomized, controlled study. J. Clin. Endocrinol. Metab..

[129] Taieb D., Lussato D., Guedj E., Roux F., Mundler O. (2006). Early sequential changes in serum thyroglobulin after radioiodine ablation for thyroid cancer: Possible clinical implications for recombinant human thyrotropin-aided therapy. Thyroid.

[130] Duntas L.H., Biondi B. (2007). Short-term hypothyroidism after Levothyroxine-withdrawal in patients with differentiated thyroid cancer: Clinical and quality of life consequences. Eur. J. Endocrinol..

[131] Ma C., Tang L., Fu H., Li J., Wang H. (2013). rhTSH-aided low-activity versus high-activity regimens of radioiodine in residual ablation for differentiated thyroid cancer: A meta-analysis. Nucl. Med. Commun..

[132] Borget I., Remy H., Chevalier J., Ricard M., Allyn M., Schlumberger M., De Pouvourville G. (2008). Length and cost of hospital stay of radioiodine ablation in thyroid cancer patients: Comparison between preparation with thyroid hormone withdrawal and thyrogen. Eur. J. Nucl. Med. Mol. Imaging.

[133] Dietlein M., Busemeyer S., Kobe C., Schmidt M., Theissen P., Schicha H. (2010). Recombinant human TSH versus hypothyroidism. Cost-minimization-analysis in the follow-up care of differentiated thyroid carcinoma. Nukl. Nucl. Med..

[134] Luster M., Felbinger R., Dietlein M., Reiners C. (2005). Thyroid hormone withdrawal in patients with differentiated thyroid carcinoma: A one hundred thirty-patient pilot survey on consequences of hypothyroidism and a pharmacoeconomic comparison to recombinant thyrotropin administration. Thyroid.

